# *Bacillus velezensis* promotes the proliferation of lactic acid bacteria and influences the fermentation quality of whole-plant corn silage

**DOI:** 10.3389/fpls.2024.1285582

**Published:** 2024-02-15

**Authors:** Yili Wang, Gangqing Ying, Zimo Zhang, Yu Tang, Yunhua Zhang, Lijuan Chen

**Affiliations:** ^1^ College of Animal Science and Technology, Anhui Agricultural University, Hefei, China; ^2^ College of Resources and Environment, Anhui Agricultural University, Hefei, China

**Keywords:** *Bacillus velezensis*, proliferation, lactic acid bacteria, whole-plant corn, silage

## Abstract

**Objective:**

This study aimed to investigate the promoting effect of a *Bacillus velezensis* (BV) strain on lactic acid bacteria (LAB) and determine its influence on the fermentation quality and aerobic stability of silage.

**Methods:**

Flat colony counting method was used to evaluate the effect of BV on the growth of LAB. Freshly harvested whole-plant corn was inoculated separately with BV and L. plantarum (LP), along with an uninoculated control group (CK), and assessed at 1, 3, 5, 7, 15, and 30 days of ensiling.

**Results:**

The results indicated that BV exhibited a proliferative effect on *Weissella confusa, Lactobacillus plantarum* L-2, and Pediococcus pentosaceus. And exhibited a more rapid pH reduction in BV-inoculated silage compared with that in CK and LP-inoculated silage during the initial stage of ensiling. Throughout ensiling, the BV and LP experimental groups showed enhanced silage fermentation quality over CK. Additionally, relative to LP-inoculated silage, BV-inoculated silage displayed reduced pH and propionic acid. BV also prolonged aerobic stability under aerobic conditions. The microbial community in BV-inoculated silage showed greater stability than that in LP-inoculated silage. Additionally, *Firmicutes* and *Lactobacillus* exhibited more rapid elevation initially in BV versus LP-inoculated silage, but reached comparable levels between the two inoculation groups in the later stage.

**Conclusion:**

In summary, BV enhanced the efficacy and aerobic stability of whole-plant corn silage fermentation by stimulating LAB proliferation.

## 1 Introduction

Whole-plant corn silage is one of the most important roughage sources worldwide (Zhang et al., 2022a). It is characterized by high yield, rich nutrient content, good palatability and digestibility, making it an indispensable basic feed source for ruminants, especially dairy cattle, in Europe and the United States, whole-plant corn silage has become a vital and widely used feed in dairy production (Khan et al., 2015; Ferraretto et al., 2018; Reed et al., 2022; Shi et al., 2022a). The application of whole-plant corn silage solves the problem of insufficient feed supply for livestock, reduces farming costs to some degree, ensures stable agricultural development, and promotes increased production and yield of ruminant livestock such as cattle and sheep (Shi et al., 2022a). Zhao et al. (2020) reported that the use of whole-plant corn silage as the only roughage in a complete mixed diet improves the growth performance and meat quality of beef sheep. Cui et al. (2022) demonstrated that whole-plant corn silage enhances rumen flora in beef cattle, which in turn improves rumen fermentation and growth in beef cattle. Silva et al. (2021) found that whole-plant corn silage increases dry matter intake and milk yield in dairy cows. Zhang et al. (2022a) showed that meat quality of beef cattle fed whole corn silage improves. The development of high-quality whole-plant corn silage is vital to increasing the proportion of grass-fed livestock production in China.

Whole-plant corn silage is a method of preserving whole corn under anaerobic conditions based on fermentation by lactic acid bacteria (LAB). LAB utilize soluble sugars and other substances in whole corn as substrates, metabolizing them to produce organic acids and, creating an acidic environment that inhibits the growth of harmful microorganisms and preserves the whole-plant corn silage for an extended period to avoid deterioration and spoilage (Pahlow et al., 2003; Wilkinson et al., 2003). Nevertheless, the production of superior quality whole-plant corn silage in practice is impeded by several factors, especially the brief stabilization period under aerobic conditions post-exposure, its tendency to undergo, secondary fermentation is one of the most critical impediments impacting the efficacy of whole-plant corn silage utilization (McDonald et al., 2010; Shi et al., 2022b). Secondary fermentation is attributed to the activation of aerobic microbes including molds, *Clostridia*, and *Enterobacteriaceae* upon exposure to oxygen, eliciting aerobic degradation and subsequent elevation of silage pH and internal temperature (Haq et al., 2021). Deleterious microorganisms present during the pre-ensiling and post-exposure periods lead to diminished whole corn silage quality, elevated mycotoxin levels in whole-plant corn silage, and adverse effects on feed intake, productivity, reproduction, livestock product quality, and mortality in livestock after feeding (Binder, 2007; Richard, 2007; Drouin et al., 2021; Chen et al., 2022). Mitigating the detrimental impacts of harmful microflora on whole-plant corn silage and ameliorating silage quality through silage additives have elicited profound research interest. Common additives comprise microbial inoculants (e.g., *Lactobacillus* and *Bacillus* spp.), enzymes (e.g., cellulase and hemicellulose), and chemical additives (e.g., formic acid and benzoic acid), among others (Muck et al., 2018). Of all the additives, microbial inoculants are the most extensively utilized primarily by LAB. These inoculants can swiftly reduce silage pH by rapid proliferation during the initial ensiling phase, suppressing deleterious acid-intolerant microbes and yielding superior quality silage (Carvalho et al., 2021; Chen et al., 2023). Nascimento Agarussi et al. (2019) used *Lactobacillus plantarum* and *Pediococcus pentosaceus* to ensile alfalfa and found that both LAB were able to enhance the fermentation quality of the silage feed. Li et al. (2021) discovered that inoculating with *L. plantarum* and *Lactobacillus buchneri* could mitigate the adverse effects of fungi on ensiled corn by changing the bacterial and fungal communities, thereby improving the fermentation quality of the corn silage. Presently, *Bacillus* spp. also garnering interest because of their capacity to hydrolyze plant cell walls, releasing soluble sugars via cellulase and hemicellulase production and facilitating LAB (Ning et al., 2017; Li et al., 2018). Concurrently, *Bacillus* can suppress undesirable microbes like molds and yeasts in the initial and post-exposure phases by generating bacteriocins. Several studies have indicated that using *B.* spp. as a silage additive can improve the fermentation quality and aerobic stability of silage feed, and have positive effects on the microbial community (Bai et al., 2020; Bonaldi et al., 2021; Zhu et al., 2022). In this study, *Bacillus velezensis* (BV) was utilized, which has demonstrated an ability to restrain harmful microbes including molds, *Escherichia* coli and yeasts in prior studies. Theoretically, BV as an inoculant can ameliorate the fermentation quality of whole corn silage, although its effects in whole corn silage application remain uninvestigated. Therefore, this study aimed to examine the impacts of this BV strain on whole-plant corn silage quality when utilized as a silage inoculant.

## 2 Materials and methods

### 2.1 Effect of BV on LAB proliferation

Flat colony counting method was used to evaluate the effect of BV on the growth of LAB. The experiment was divided into three groups: the blank group (inoculated with 2% BV at a concentration of 1.0 × 10^6^ cfu (colony forming units)/g in De Man, Rogosa and Sharpe (MRS) liquid medium), the control groups (inoculated with 1% LAB at a concentration of 1.0 × 10^6^ cfu/g in MRS liquid medium), and the experimental groups (inoculated with 2% BV at a concentration of 1.0 × 10^6^ cfu/g and 1% LAB at a concentration of 1.0 × 10^6^ cfu/g in MRS liquid medium). After each group was cultured for 12 h, equal amounts of bacterial suspensions were taken and diluted to 10^−5^. Then, 15 μL of the diluted suspensions was spread onto the MRS solid medium and incubated for 12 h. The bacterial colony counts in the culture dishes were used to calculate the cfu/g of MRS medium. For this experiment, three types of LAB were selected: *Weissella confusa*, *L. plantarum* L-2, and *P. pentosaceus*. These LABs were isolated, identified, and preserved from whole-plant corn silage material. The BV used in the experiment was isolated, identified, and preserved from the environment in the laboratory. The MRS, Luria-Bertani culture media, and agar were purchased from HaiBo Biotechnology Co., Ltd (Qingdao).

### 2.2 Preparation of silage

After whole-plant corn (National High tech Agricultural Park of Anhui Agricultural University, 31° 58′ N, 117° 24′ E) was harvested at the milk-ripe stage, it was chopped into 2–3 cm lengths and thoroughly mixed before being randomly sampled for ensiling. The experiment included one control group and two treatment groups: control (CK) group (1% saline added), BV-inoculated silage, with the addition of 1% BV at a concentration of 1.0 × 10^6^ cfu/g FM, and LP-inoculated silage, with the addition of 1% *L. plantarum* (LP) at a concentration of 1.0 × 10^6^ cfu/g FM. Each group had 3 replicates. Silage was carried out using silage bags (250 × 300 mm, Hefei Xi Yue Biological Co., Ltd., Hefei), with 400 g of material per bag. The bags were stored at room temperature in darkness. Samples were collected at room temperature (25 ± 2°C) for analysis on days 1, 3, 5, 7, 15, and 30 of ensiling (Each days had 3 replicates). The LP, a commercial *L. plantarum* strain (Guangzhou Weiyuan Biotech Co., Ltd, Guangzhou, China), has a viable count of 50 billion cfu/g FM.

### 2.3 Chemical composition analysis

Ten grams of ensiled sample was mixed with 90 mL distilled water and extracted at 4°C for 12 hours. The pH of the filtrate was measured using a pH meter (Mettler Toledo). Approximately 10 mL of the filtrate was centrifuged (4500× g, 15 minutes, 4°C), and the supernatant was analyzed for lactic acid (LA), acetic acid (AA), propionic acid (PA), and butyric acid (BA) contents by using high-performance liquid chromatography (HPLC). An Agilent TC-C18 column (250 nm × 4.6 nm, 5 μm) was used, with acetonitrile as mobile phase A and 0.01 mol/L potassium dihydrogen phosphate (pH 2.70) as mobile phase B at a ratio of 3:97. The flow rate was set to 0.6 mL/min, and UV detection was performed at a wavelength of 210 nm. The column temperature was maintained at room temperature (Wang et al., 2019). Ammonia nitrogen was determined following the method of Broderick and Kang (1980).

The content of dry matter (DM) were analyzed following the AOAC method. Water-soluble carbohydrates (WSCs) were measured using anthrone-sulfuric acid colorimetriy to determine soluble sugars, in accordance with AOAC (1990). Crude protein (CP) was determined using the Kjeldahl method with an automated nitrogen analyzer. Neutral detergent fiber (NDF) and acid detergent fiber (ADF) were determined using a fiber analyzer on the basis of the analysis system outlined by Van Soest et al. (1991).

### 2.4 Aerobic stability

After 30 days of ensiling whole-plant corn, the silage bags were opened. The contents were transferred into plastic containers (500 mL) and placed in a room maintained at 25°C, and the containers were left uncovered. At intervals of 30 minutes, the temperature at the center of the silage was measured using a digital temperature probe until the sample temperature exceeded the ambient temperature by 2°C (Chen et al., 2016).

### 2.5 Bacterial community analysis

Total genomic DNA of bacteria on the surface of fresh and silage maize feeds at time points 1, 3, 5, 7, 15, and 30 days of fermentation was extracted utilizing a DNA isolation kit (D4015, Omega, Inc., USA). Polymerase Chain Reaction (PCR) amplification of the full-length 16S rRNA gene of bacteria was conducted using forward primer 343F (TACGGRAGGCAGCAG) and reverse primer 798R (AGGGTATCTAATCCT).

For PCR amplification, the total reaction volume was 25 μL, comprising 25 ng of template DNA, 12.5 μL of PCR premix, 2.5 μL of each primer, and PCR-grade water to adjust the volume. The PCR amplicons were purified by AMPure XT beads (Beckman Coulter Genomics, Danvers, MA, USA) and quantified using Qubit (Invitrogen, USA). Amplicon pools were prepared for sequencing. The size and quantity of the libraries were assessed on Agilent 2100 Bioanalyzer (Agilent, USA) and an Illumina library quantification kit (Kapa Biosciences, Woburn, MA, USA), respectively. Sequencing was performed on an Illumina NovaSeq PE250 platform. The samples were sequenced on an Illumina NovaSeq platform. Paired-end reads were assigned based on unique sample barcodes and truncated by cutting off barcode and primer sequences. Paired-end reads were merged using FLASH. Raw reads were subjected to quality filtering under specific conditions by fqtrim (v0.94) to obtain high-quality, clean sequences. Chimeric sequences were filtered using Vsearch software (v2.3.4). Feature tables and feature sequences were obtained after iterative processing with DADA2. Alpha diversity and beta diversity were calculated by QIIME2, and the same number of sequences was randomly selected by reducing the number of sequences to the minimum of some samples. Bacterial taxa were classified based on relative abundance (X number of bacteria/total number). Species-annotated sequence comparisons were performed using Blast software, and the comparative databases utilized were SILVA and NT-16S. Linear discriminant analysis effect size analysis (LEfSe) was employed to identify communities or species with significant differences among the three groups. The sequence data from this study have been deposited in the NCBI database under Accession No. PRJNA1013177.

### 2.6 Statistical analyses

Single-factor ANOVA was employed to analyze the microbial populations, chemical composition, and fermentation quality data of fresh and ensiled whole-plant corns to assess the efficacy of LAB inoculants. Duncan’s multiple range test was utilized to assess differences among means. *P* < 0.05 was considered statistically significant. The analysis was conducted using IBM SPSS Statistics 26.0 (SPSS, Inc., Chicago, IL).

## 3 Results

### 3.1 Effect of BV on LAB proliferation

**Figure 1:**
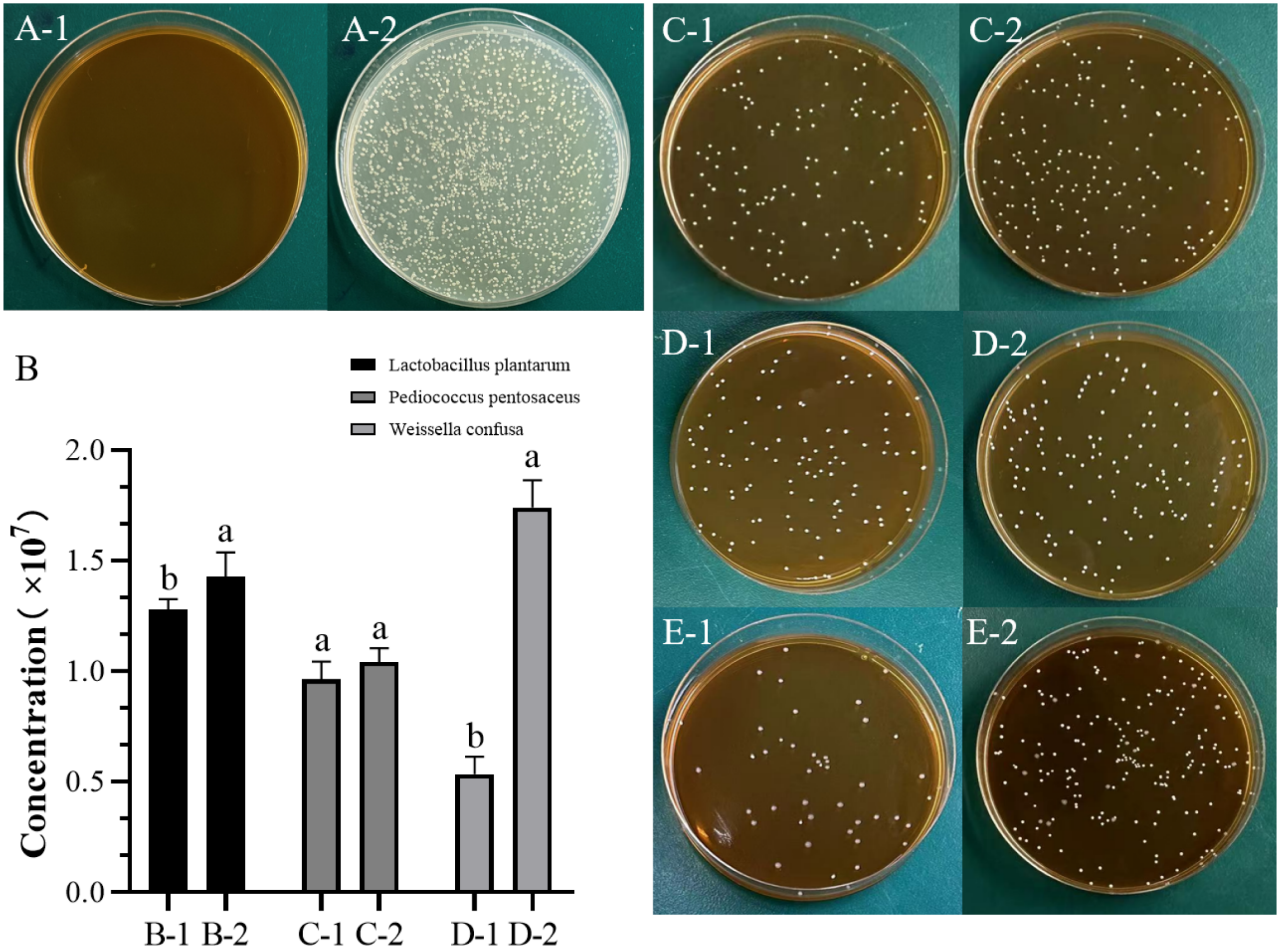
Promoting effect of *Bacillus velezensis* on lactic acid bacteria. Different lowercase letters indicate significant differences (*P* < 0.05). **(A)** Picture of *B. velezensis* cultured on Luria-Bertani and MRS plates for 12 hours. (A-1) MRS medium. (A-2) Luria-Bertani medium. **(B)** Concentration column chart of each milliliter of bacterial liquid after 12 hours of cultivation. **(C)** *L. plantarum* L-2 group. (C-1) Control group without *B.velezensis*. (C-2) Experimental group with *B. velezensis* added. **(D)** *P. pentosaceus* group. (D-1) Control group without *B. velezensis*. (D-2) Experimental group with *B. velezensis* added. **(E)** *W. confusa* group. (E-1) Control group without *B. velezensis*. (E-2) Experimental group with *B. velezensis* added. MRS, De Man, Rogosa and Sharpe. (*L. plantarum* L-2 and LP are two different strains of *L. plantarum*.).

In this experiment, BV was cultured in the MRS medium as a blank group, but it did not grow after 12 hours in MRS medium (**Figure 1A-1**). However, it exhibited normal growth on the Luria-Bertani agar media (**Figure 1A-** **2**). BV was mixed cultured with different LABs for 12 hours, followed by subsequent cultivation on MRS solid medium for an additional 12 hours (**Figure 1B**). The results indicated that this bacterium exhibited a promotive effect on the proliferation of *L. plantarum* L-2 (**Figure 1C**), *P. pentosaceus* (**Figure 1D**), and *W. confusa* (**Figure 1E**). In Particular, a significant enhancement was found on the growth of *L. plantarum* L-2 and *W. confusa* (*P* < 0.05), whereas the promotion of *P. pentosaceus* was not significant (*P* > 0.05).

### 3.2 Chemical composition and fermentation characteristic analysis

As shown in **Table 1**, the DM content of the whole-plant corn material was 27.49%. On the basis of DM, the CP, EE, crude ash, NDF, ADF, and WSC contents were 10.60%, 7.85%, 3.62%, 26.57%, 14.60%, and 8.15%, respectively.

**Table 1:** Chemical composition of fresh samples.

Items	Fresh matter
DM, % FM	27.49±0.30
Ash, % DM	3.62±0.06
CP, % DM	10.60±0.04
EE, % DM	7.85 ± 0.37
WSC, % DM	8.15 ± 0.15
NDF, % DM	26.57±0.54
ADF, % DM	14.60±0.40

DM, dry matter; WSC, water-soluble carbohydrates; CP, crude protein; EE, ether extract; NDF, neutral detergent fiber; ADF, acid detergent fiber.

**Table 2** shows that ensiling time significantly reduced the DM content (*P* < 0.05), and the additives did not (P > 0.05). Moreover, the additives and ensiling time did not significantly affect the CP content (*P* > 0.05). Ensilage time did not have a significant effect on the NDF and ADF contents (*P* > 0.05). The NDF content in BV- and LP-inoculated silages was lower than that of CK at days 1, 3, and 30 (*P* < 0.05), with no significant difference between the two test groups (*P* > 0.05). At day 5, the LP-inoculated silage had a lower NDF content than CK (*P* < 0.05), whereas the BV-inoculated silage did not significantly differ from the other groups (*P* > 0.05). The ADF content in the BV-inoculated silage was lower than those in CK and LP-inoculated silage at days 1, 5, 7, and 15 (*P* < 0.05).

**Table 2:** Effect of *Bacillus velezensis* on the chemical composition of whole-plant corn silage.

	Treatments(T)	Storage period (D)						SEM	P-value		
		1	3	5	7	15	30		T	D	T×D
DM, % FM	CK	33.60a	33.59a	32.22b	31.39c	30.87d	27.71e	0.003	0.992	<0.001	0.986
	BV	33.63a	33.56a	32.31b	31.43c	30.75d	27.69e				
	LP	33.61a	33.62a	32.30b	31.41c	30.35d	27.68e				
CP, % DM	CK	8.21	7.67	7.54	7.49	7.48	7.42	0.007	0.315	0.225	0.969
	BV	8.57	7.92	7.8	7.78	7.74	7.68				
	LP	8.51	7.93	7.92	7.84	7.75	7.64				
NDF, % DM	CK	27.98A	27.89A	27.14A	27.26	27.10	27.66A	0.008	<0.001	0.403	0.894
	BV	26.63B	26.26B	26.34AB	26.43	26.06	26.12B				
	LP	26.77aB	26.44abB	26.15abB	25.99b	26.08ab	26.27abB				
ADF, % DM	CK	17.95A	17.84	17.94A	17.61A	17.66A	17.63	0.006	<0.001	0.631	0.775
	BV	16.97B	16.9	16.4B	16.43B	16.30B	16.87				
	LP	17.77A	17.69	17.49A	17.33A	17.41A	17.42				
Ammonia-N % TN	CK	2.19dA	3.37cA	3.97bcA	3.37cA	4.04abA	4.27aA	<0.001	0.001	<0.001	0.476
	BV	1.88cB	2.65bB	2.85bB	2.58bB	2.86bB	3.48aB				
	LP	1.99dB	2.74bcAB	2.98bcAB	2.58cB	3.08abB	3.46aB				
WSC, % DM	CK	5.08a	2.93bB	1.35cC	1.32cdC	1.28cdC	1.17dB	0.002	0.381	<0.001	0.847
	BV	5.09a	4.39bA	2.49cA	2.13dA	1.94eA	1.70fA				
	LP	5.04a	2.70bC	2.11cB	1.86dB	1.72eB	1.60fA				

Differences marked with different uppercase letters in the same column are significant (P < 0.05), and differences marked with different lowercase letters in the same row are significant (P < 0.05). BV, *Bacillus velezensis* added; LP, Lactobacillus plantarum added.

With the increase in ensiling days, the NH_3_-N content in all groups increased (*P* < 0.05). Compared with CK, the LP-inoculated silage had reduced NH_3_-N content on days 1, 7, 15, and 30 (*P*< 0.05), whereas the BV-inoculated silage showed a decrease in NH_3_-N content in all ensiling days (*P* < 0.05). No significant difference was found in the NH_3_-N content between the LP and BV-inoculated silages (*P* > 0.05).

As the ensiling time progressed, the WSC content exhibited a decreasing trend in all groups (*P* < 0.05). LP and BV inoculations reduced the losses of WSC in the silage compared with CK (*P* < 0.05). The BV-inoculated silage had the highest WSC content, higher than the LP-inoculated silage on days 3, 5, 7, and 15 (*P* < 0.05).

In **Table 3**, the pH values of the three groups rapidly decreased during the ensiling process and exhibited a significantly decreasing trend (*P* < 0.05). The pH values of the BV-inoculated silage were lower than those of the control at all observed time points (*P* < 0.05). Specifically, the pH values of the BV-inoculated silage were lower than those of the LP-inoculated silage on days 1, 3, 5, 7, and 30. Meanwhile, the pH values of the LP-inoculated silage were lower than those of CK on days 1, 3, 7, and 15 (*P* < 0.05). The LP-inoculated silage exhibited the highest LA content, significantly surpassing that of the CK group throughout the entire ensiling period (*P* < 0.05). In the BV-inoculated silage, the LA content was notably higher than that of the CK group during the initial 7 days of ensiling, subsequently dropping on day 15 (*P* < 0.05) and showing no significant difference from that of the CK group on day 30 (*P* > 0.05). The AA content in the LP-inoculated silage was lower than that of CK during the first 3 days of ensiling, but it increased as the ensiling time progressed (*P* < 0.05). By contrast, the BV-inoculated silage exhibited lower AA content than CK on day 1 (*P* < 0.05), followed by higher levels from day 3 to day 15 (*P* < 0.05), with no significant difference from the control on day 30 (*P* > 0.05). For PA content, the LP and BV-inoculated silages maintained lower levels than CK at all observed time points (*P* < 0.05). Moreover, the BV-inoculated silage had the lowest PA content. Overall, inoculation with exogenous silage inoculants increased the LA and AA contents while reducing the PA content in the ensiled maize feed. No detectable BA was observed in any of the groups during the entire ensiling period.

**Table 3:** Effect of *Bacillus velezensis* on the quality of whole-plant corn silage.

	Treatments(T)	Storage period (D)						SEM	P-value		
		1	3	5	7	15	30		T	D	T×D
pH	CK	5.06aA	4.51bA	4.34cA	4.20dA	4.00eA	3.89fA	0.052	0.122	<0.001	0.687
	BV	4.66aC	4.30bC	4.00cB	3.80dC	3.86dB	3.70eB				
	LP	4.96aB	4.38bB	4.03cA	3.99cB	3.89dB	3.80dA				
LA% DM	CK	1.98fB	3.08eC	3.81dB	4.99cC	8.49bB	9.24aB	0.004	0.021	<0.001	0.350
	BV	2.17eA	5.59dA	5.59dA	8.52bA	8.23cC	9.29aB				
	LP	2.13eA	3.88dB	5.52cA	7.81bB	10.01aA	10.00aA				
AA, % DM	CK	1.21bA	1.05cB	0.41fB	0.56eC	0.70dC	1.86aB	<0.001	0.008	<0.001	<0.001
	BV	1.11cdB	1.15cA	1.03dA	1.38bA	0.77eB	1.96aB				
	LP	1.12bB	0.99cC	0.99cA	0.80dB	1.12bA	3.06aA				
PA, % DM	CK	2.56fA	3.53dA	4.44cA	3.29eB	5.98bA	9.11aA	0.003	<0.001	<0.001	<0.001
	BV	1.92eC	2.94dB	3.26cC	3.79bA	3.99bC	7.06aC				
	LP	2.30eB	2.91dB	3.59cB	3.55cB	4.95bB	7.63aB				
BA, % DM	CK	ND	ND	ND	ND	ND	ND				
	BV	ND	ND	ND	ND	ND	ND				
	LP	ND	ND	ND	ND	ND	ND				

Differences marked with different uppercase letters in the same column are significant (P < 0.05), and differences marked with different lowercase letters in the same row are significant (P < 0.05). LA, lactic acid; AA, acetic acid; PA, propionic acid; BA, butyric acid. BV, *Bacillus velezensis* added; LP, Lactobacillus plantarum added.

### 3.3 Aerobic stability

**Figure 2:**
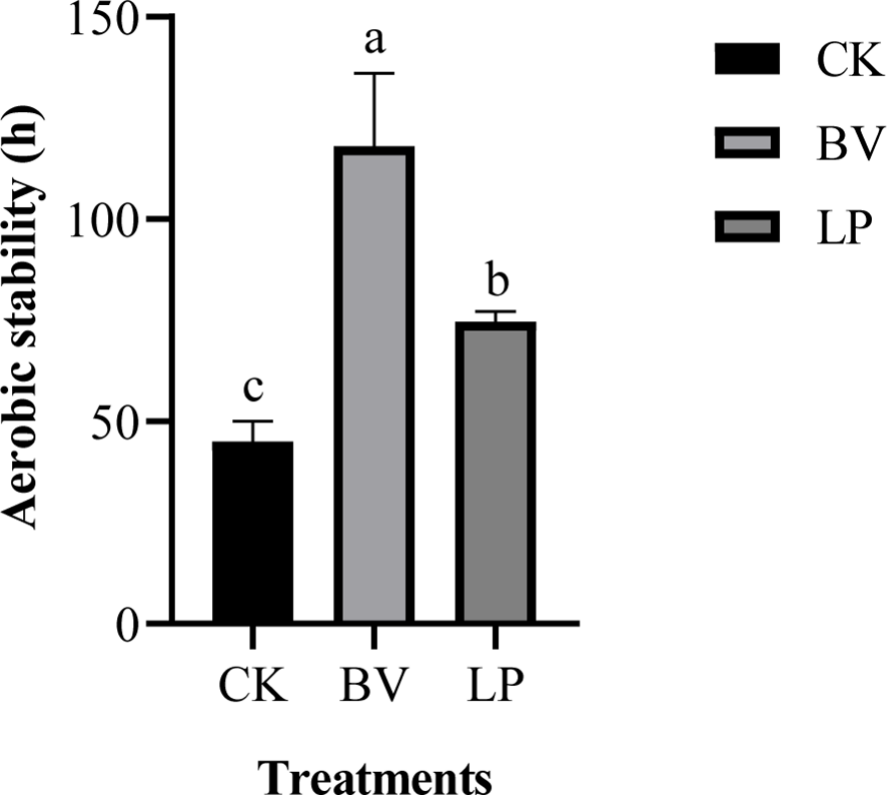
Aerobic stability of whole-plant corn silage. Different lowercase letters indicate significant differences (*P* < 0.05). CK: control group; BV, *Bacillus velezensis* added; LP, *Lactobacillus plantarum* added.

The aerobic stability of whole-plant corn silage is depicted in **Figure 2**. The aerobic stability times for CK, the BV-inoculated silage, and the LP-inoculated silage were 45, 118, and 73 hours, respectively. The BV-inoculated silage exhibited the longest aerobic stability time, which was higher than CK (*P* < 0.05). Moreover, the LP-inoculated silage had higher aerobic stability time than CK (*P* < 0.05).

### 3.4 Bacterial community composition and diversity in whole-plant corn silage

**Figure 3:**
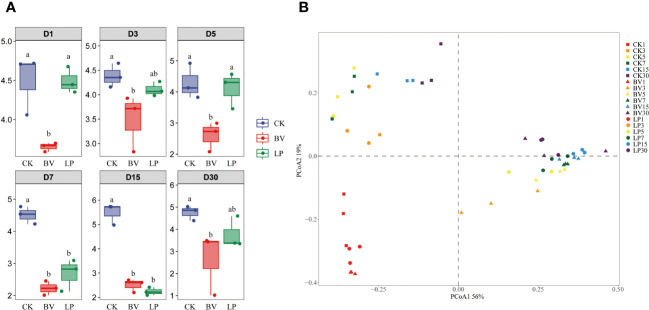
Bacterial community diversities and compositions in whole-plant corn silage during ensiling. Different lowercase letters indicate significant differences (*P* < 0.05). **(A)** Variations in community alpha-diversities (Shannon index). **(B)** Community dissimilarities in different groups and fermentation times, calculated using principal coordinate analysis (PCoA). BV, *Bacillus velezensis* added; LP, *Lactobacillus plantarum* added.

The bacterial community diversity of whole-plant corn silage is illustrated in **Figure 3**. During the ensiling process, the alpha diversities (Shannon index) of the BV and LP experimental groups was lower than that of CK (**Figure 3A**). In accordance with beta diversity (principal coordinate analysis, PCoA), significant differences and consistent changes in bacterial community composition were observed in whole-plant corn silage at different fermentation stages (**Figure 3B**). BV and LP did not exhibit clear separation from CK on day 1 of ensiling. However, on other ensiling days, both experimental groups were distinct from CK, whereas no clear separation was observed between the two experimental groups.

**Figure 4:**
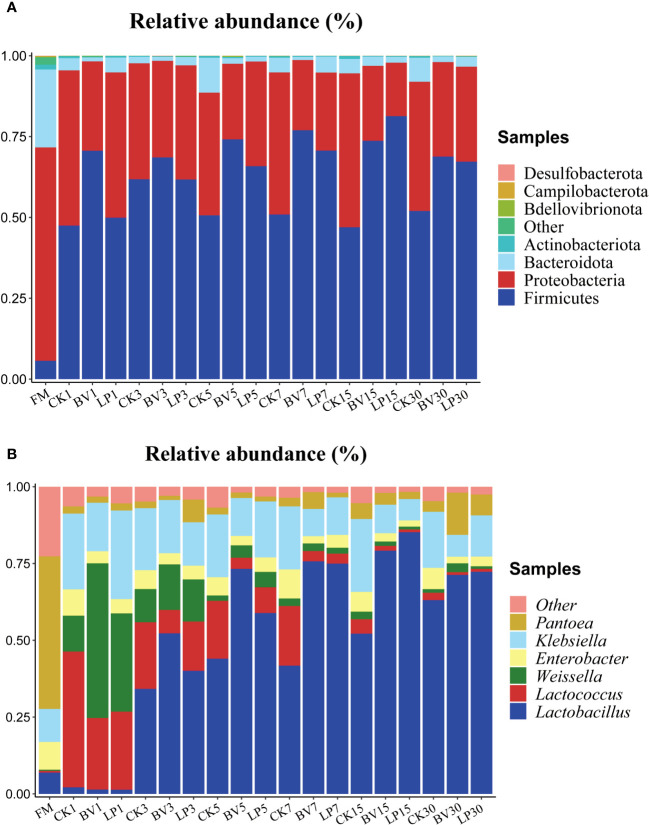
Bacterial community composition and succession of whole-plant corn silage. **(A)** Phylum level. **(B)** Genus level. BV, *Bacillus velezensis* added; LP, *Lactobacillus plantarum* added.

The bacterial community composition of whole-plant corn silage at the phylum and genus levels is presented in **Figure 4**. As shown in **Figure 4A**, the main epiphytic bacteria at the phylum level before ensiling were *Proteobacteria* and *Bacteroidetes*, whereas *Firmicutes* had a relatively lower proportion. After ensiling, the proportions of *Proteobacteria* and *Bacteroidetes* decreased, whereas that of *Firmicutes* increased. On different ensiling days, the abundance of *Firmicutes* in the BV-inoculated silage was higher than that in CK, whereas the abundance of *Bacteroidetes* was lower in the experimental groups than in CK. In the LP-inoculated silage, the proportion of *Firmicutes* showed no significant change compared with that in CK on day 3, but it was higher on other ensiling days. Meanwhile, the abundance of *Bacteroidetes* was lower than that in CK on all ensiling days.

As shown in **Figure 4B**, the predominant genus in fresh samples at the genus level was *Pantoea*. On day 1 of ensiling, the dominant genus in the CK and experimental groups were *Lactococcus*, *Weissella*, and *Klebsiella*. As fermentation progressed, the abundance of *Lactobacillus* gradually increased and became dominant, whereas the proportions of *Weissella*, *Lactococcus*, and *Klebsiella* decreased. After 3 days of ensiling, *Lactobacillus* was higher in the two experimental groups than in CK. Moreover, it was lower in the LP-inoculated silage than in the BV-inoculated silage from day 1 to day 5 and higher from day 7 to day 30.

**Figure 5:**
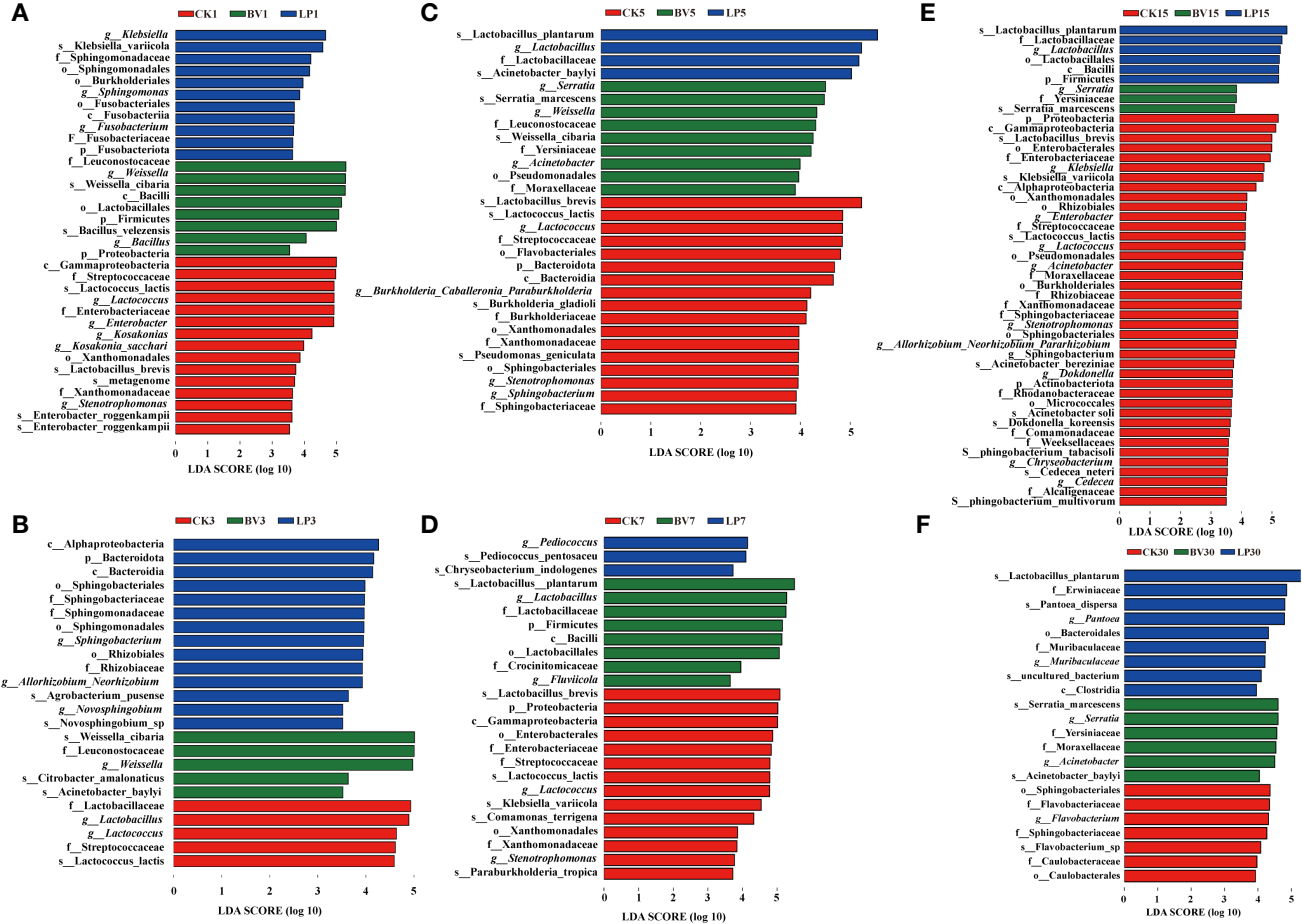
Identification of the communities or species with significant differences among three groups. BV, *Bacillus velezensis* added; LP, *Lactobacillus plantarum* added. **(A)** after 1 d of ensiling; **(B)** after 3 d of ensiling; **(C)** after 5 d of ensiling; **(D)** after 7 d of ensiling; **(E)** after 15 d of ensiling; **(F)** after 30d of ensiling.

LEfSe analysis was performed to explore the differences in bacterial communities among the three groups, as shown in **Figure 5**. On day 1 of ensiling (**Figure 5A**), *Proteobacteria*, *Lactococcus*, and *Enterobacter* were higher in CK, *Weissella* was higher in the BV-inoculated silage, and *Klebsiella* was higher in the LP-inoculated silage. On day 3 of ensiling (**Figure 5B**), *Lactobacillus* and *Lactococcus* were higher in CK, *Weissella* was higher in the BV-inoculated silage, and *Bacteroides* and *Sphingobacterium* were higher in the LP-inoculated silage. On day 5 of ensiling (**Figure 5C**), *Lactobacillus*, *Lactococcus*, and *Streptococcus* were more abundant in CK, *Weissella* was significantly higher in the BV-inoculated silage, and *Lactobacillus* was higher in the LP-inoculated silage. On day 7 of ensiling (**Figure 5D**), CK remained to have higher *Lactobacillus brevis*, *Lactococcus* and demonstrated an increase in *Proteus*. *Pediococcus* was higher in the LP-inoculated silage, whereas *Bacillus* and *Lactobacillus* were enriched in the BV-inoculated silage. On day 15 of ensiling (**Figure 5E**), *Proteus* was higher in CK, *Serratia* was higher in the BV-inoculated silage, and *Lactobacillus* and *Lactococcus* were higher in the LP-inoculated silage. On day 30 of ensiling (**Figure 5F**), *Flavobacterium* and *Sphingobacterium* were higher in CK, *Lactobacillus* and *Bacteroides* were enriched in the LP-inoculated silage, and *Serratia* and *Acinetobacter* were higher in the BV-inoculated silage.

## 4 Discussion

In general, LAB are common inoculants used for ensiling feedstock (Muck et al., 2018; Mu et al., 2020; Yang et al., 2021). They can rapidly lower the pH of ensiled materials and improve fermentation quality (Chen et al., 2023). However, in recent years, studies found that due to their ability to produce cellulases, hemicellulases, and amylases, *B.* spp. were used as exogenous inoculants for silage fermentation (Bonaldi et al., 2021), to enhance the fermentation efficiency of plant materials in ensiling. Several reports (Bai et al., 2020; Bonaldi et al., 2021; Bai et al., 2022a) indicated that the use of *B.* spp. as silage inoculants can improve the fermentation quality and reduce the loss of nutritional value in silage. As one of the strains of *Bacillus*, *B. velezensis* possesses potential probiotic properties (Khalid et al., 2021). It can promote the growth and reproduction of *W*. *confusa*, *P. pentosaceus*, and *L. plantarum* L-2 in ensiled whole-plant corn feedstock. Furthermore, the whole-plant corn ensiled with BV treatment exhibited significantly lower pH values, especially in the initial 7 days of ensiling, than CK and the LP-inoculated silage. This finding indicated that BV has a rapid pH-lowering effect on whole-plant-corn-ensiled feedstock due to its promotion of LAB proliferation. This result indicated that BV rapidly reduces the pH of whole-plant corn silage in the early stage of ensiling by promoting the proliferation of LAB. LP also contributes to lowering pH during corn ensiling, but its pH-lowering effect, especially during the first 7 days of ensiling, is weaker than that of BV at the same dosage, further confirming the role of BV in promoting the growth of LAB. In the early stage of ensiling, the pH of silage inoculated with LP was significantly lower than CK. As ensiling progressed, the pH of CK decreased rapidly, and by day 30 of ensiling, the difference in pH between CK and the LP-inoculated silage became insignificant. This finding can be attributed to the higher initial population of exogenous LP in the whole corn plant during the early ensiling stages in comparison to CK. As ensiling progressed, the epiphytic LAB in CK gradually proliferate and produce organic acids that lower the pH of the ensiling environment. In the late fermentation stage, microbial activity was inhibited in all groups due to low pH values, and finally, pH reached a stable state on day 30.

During ensiling fermentation, LABs utilize carbohydrates to produce LA. In this study, the silage inoculated with LP exhibited lower LA concentration than the BV-inoculated silage during the first 7 days, and then it reached its highest concentration on days 15 and 30. This finding suggested that the LP originating from exogenous environment require some time to adapt to the ensiling substrate in the early stages of fermentation. As the ensiling time was extended, LP gradually acclimated to the environment of whole-plant corn silage. Meanwhile, the LA concentration in the BV-inoculated silage was higher than that in CK on days 1, 3, 5, 7, and 30. This finding may be attributed to the rapid depletion of oxygen by BV during the fermentation process, thus creating an anaerobic environment that favors the growth of LABs and promoting their proliferation. In this experiment, the use of LP suppressed the generation of AA in the early stage, but in the later stages, the AA was significantly higher than that in CK. *L. plantarum* has traditionally been considered a homofermentative LAB, that mainly produces LA. However, recent studies suggested that *L. plantarum* is a facultative heterofermentative LAB. In addition to utilizing glucose to produce LA, *L. plantarum* produces phosphoketolase, an enzyme that can utilize pentose to produce LA and some AAs (Muck et al., 2018). Furthermore, some researchers pointed out that when using *L. plantarum* for ensiling, a low AA content in the early stages may result in weakened inhibition of yeast activity, leading to aerobic spoilage and subsequently causing an increase in AA content in the later stages (Lynch et al., 2012). Both possibilities mentioned above could potentially lead to increased AA content. However, the specific reason for the increased AA content in the LP group in the present experiment warrants further investigation. The inoculation of BV resulted in higher AA content than that in CK during 3–30 days of ensiling. This finding is in line with the findings of Bai et al. (2022b) and Xu et al. (2018), that is inoculation of *Bacillus* is beneficial for increasing the AA content in silage. Similar results suggested that a moderate amount of AA can enhance the aerobic stability of silage (Danner et al., 2003). In addition, the BV-inoculated silage showed a higher AA content than the LP-inoculated silage in the first 7 days of ensiling, possibly due to the faster adaptation of BV to the ensiling environment than the exogenous LP. Moreover, in terms of aerobic stability, the BV-inoculated silage had the significantly longest time (118 hours) before a temperature increase of 2°C. This finding can be attributed to the antimicrobial capabilities of BV. Tabbene et al. (2009) indicated that *B. velezensis* can produce various antifungal compounds that have antibacterial activity against *E. coli*, *Listeria monocytogenes*, and *Fusarium oxysporum*. Fazle Rabbee and Baek (2020) indicated that genome sequencing of *B. velezensis* revealed the presence of numerous biosynthetic gene clusters that encode enzymes responsible for synthesizing various antifungal compounds. Cao et al. (2018) found that two strains of *B. velezensis* exhibited antagonistic effects against *F. oxysporum*. The inhibitory effect of BV on molds can reduce the occurrence of aerobic spoilage. In LP group, LP may potentially achieve this by preserving the abundance of *Lactobacilli* in the late stages of ensiling, thereby maximizing DM and CP retention, minimizing protein loss, and maintaining an acidic environment in the late fermentation stage to suppress the growth of acid-intolerant bacteria, thereby improving aerobic stability. Some studies showed that *L. plantarum* can enhance the aerobic stability of silage (Mu et al., 2020; Mu et al., 2021). However, contrasting findings suggested that it failed to improve aerobic stability and may have even reduce it, causing aerobic spoilage (Keshri et al., 2018; Wu et al., 2022). The mechanism behind LP increasing the aerobic stability remains to be explored.

In this experiment, the use of BV resulted in decreased the ADF and NDF contents compared with those in CK, along with a reduction in the ammonia nitrogen content in the feed. This finding may be due to the antibacterial ability of BV, which can inhibit the decomposition of protein by spoilage microorganisms, thereby reducing ammonia nitrogen and aerobic spoilage. Meanwhile, the ammonia nitrogen content in the LP group was also lower than that in CK, demonstrating that the LP used in this experiment has the ability to inhibit aerobic spoilage of corn silage and reduce protein decomposition and further confirming why LP can improve aerobic stability. Soluble sugars are one of the major components of plant silage fermentation (Liu et al., 2023). In the present study, the soluble sugar content in silage feed inoculated with BV and LP was higher than that in CK. Inoculation with LP reduced the loss of soluble sugars, whereas the BV-inoculated silage demonstrated an increase. This phenomenon is possibly due to the enzymatic action of cellulases, hemicellulases, and amylases, which all hydrolyze carbohydrates from plant cell walls, thereby releasing soluble sugars for fermentation (Bonaldi et al., 2021).

Silage feeds with higher fermentation quality typically exhibit lower α-diversity (Bai et al., 2021). In the present study, inoculation with BV and LP reduced α-diversity at various stages of silage fermentation. The PCoA plot revealed that the BV-inoculated silage significantly separated from CK after day 3 of fermentation, whereas separation from the LP-inoculated silage was not observed until day 5. This finding indicated that the effect of BV inoculation on bacterial community was similar and superior to those of LP and CK, respectively. This finding aligns with the results of Bai et al. (2022a) and Xu et al. (2021). *Lactococcus*, *Weissella*, and *Klebsiella* are commonly found in the early stages of silage fermentation. As the fermentation progressed and the pH of the feed decreased, *Klebsiella*, *Lactococcus*, and *Weissella* gradually decreased in all groups, whereas *Lactobacillus* became the dominant genus. This trend is consistent with the findings of Zhao et al. (2021); Zhang et al. (2022b), and Xu et al. (2021). The presence of microorganisms in silage feed serves as a crucial indicator of its quality. Inoculating BV can optimize microbial community within corn silage, thereby enhancing the production of high-quality silage. LEfSe analysis was employed to further investigate the differences in bacterial composition among CK, the LP-inoculated silage, and the BV-inoculated silage. The results revealed that on days 1 and 7 of silage fermentation, the abundance of *Lactobacillus*, *Bacillus*, and on day 5, the abundance of *Weissella* was higher in the BV-inoculated silage than in the LP and CK groups. In addition, the increased in LAB, especially *Weissella*, in the BV-inoculated silage suggested that BV promotes the proliferation of LAB not only in liquid media but also during the fermentation process. This phenomenon was previously observed in various studies without substantial attention (Bai et al., 2020; Zhu et al., 2022; Wang et al., 2023).

## 5 Conclusion

BV demonstrates superior effects in rapidly lowering the pH of silage feed by promoting the proliferation of LAB, enhancing the aerobic stability of the silage feed under aerobic stress conditions, and leading to a more stable microbial community structure. BV enhances the fermentation quality of whole-plant corn silage.

## Data availability statement

The datasets presented in this study can be found in online repositories. The names of the repository/repositories and accession number(s) can be found below: BioProject accession number: PRJNA1013177.

## Author contributions

YW: Writing – original draft, Writing – review & editing. GY: Writing – original draft. ZZ: Writing – review & editing. YT: Writing – review & editing. YZ: Writing – review & editing. LC: Writing – review & editing.

## References

[B1] AOAC (1990). Official methods of analysis. 15th Edn (Arlington: Association of Official Analytical Chemists).

[B2] Bai J. Ding Z. Ke W. Xu D. Wang M. Huang W.. (2021). Different lactic acid bacteria and their combinations regulated the fermentation process of ensiled alfalfa: ensiling characteristics, dynamics of bacterial community and their functional shifts. Microb. Biotechnol. 14 (3), 1171–1182. doi: 10.1111/1751-7915.13785 33666350 PMC8085944

[B3] Bai J. Ding Z. Su R. Wang M. Cheng M. Xie D.. (2022b). Storage temperature is more effective than lactic acid bacteria inoculations in manipulating fermentation and bacterial community diversity, co-occurrence and functionality of the whole-plant corn silage. Microbiol. Spectr. 10 (2), e0010122. doi: 10.1128/spectrum.00101-22 35343767 PMC9045155

[B4] Bai J. Franco M. Ding Z. Hao L. Ke W. Wang M.. (2022a). Effect of *Bacillus amyloliquefaciens* and *Bacillus subtilis* on fermentation, dynamics of bacterial community and their functional shifts of whole-plant corn silage. J. Anim. Sci. Biotechnol. 13 (1), 7. doi: 10.1186/s40104-021-00649-0 34991716 PMC8739699

[B5] Bai J. Xu D. Xie D. Wang M. Li Z. Guo. X. (2020). Effects of antibacterial peptide-producing *Bacillus subtilis* and *Lactobacillus buchneri* on fermentation, aerobic stability, and microbial community of alfalfa silage. Bioresour Technol. 315, 123881. doi: 10.1016/j.biortech.2020.123881 32731157

[B6] Binder E. M. (2007). Managing the risk of mycotoxins in modern feed production. Anim. Feed Sci. Technol. 133, 149–166. doi: 10.1016/j.anifeedsci.2006.08.008

[B7] Bonaldi D. S. Carvalho B. F. Ávila C. Silva. C. F. (2021). Effects of *Bacillus subtilis* and its metabolites on corn silage quality. Lett. Appl. Microbiol. 73 (1), 46–53. doi: 10.1111/lam.13474 33756025

[B8] Broderick G. A. Kang J. H. (1980). Automated simultaneous determination of ammonia and total amino acids in ruminal fluid and in *vitro* media. J. Dairy Sci. 63, 64–75. doi: 10.3168/jds.S0022-0302(80)82888-8 7372898

[B9] Cao Y. Pi H. Chandrangsu P. Li Y. Wang Y. Zhou H.. (2018). Antagonism of two plant-growth promoting *Bacillus velezensis* isolates against *Ralstonia solanacearum* and *Fusarium oxysporum* . Sci. Rep. 8 (1), 4360. doi: 10.1038/s41598-018-22782-z 29531357 PMC5847583

[B10] Carvalho B. F. Sales G. F. C. Schwan R. F. Ávila C. L. S. (2021). Criteria for lactic acid bacteria screening to enhance silage quality. J. Appl. Microbiol. 130, 341–355. doi: 10.1111/jam.14833 32869919

[B11] Chen L. Guo G. Yuan X. Zhang J. Li J. Shao T. (2016). Effects of applying molasses, lactic acid bacteria and propionic acid on fermentation quality, aerobic stability and in *vitro* gas production of total mixed ration silage prepared with oat-common vetch intercrop on the Tibetan Plateau. J. Sci. Food Agric. 96 (5), 1678–1685. doi: 10.1002/jsfa.7271 25996908

[B12] Chen L. Wang Y. Li X. MacAdam J. W. Zhang. Y. (2023). Interaction between plants and epiphytic lactic acid bacteria that affect plant silage fermentation. Front. Microbiol. 14. doi: 10.3389/fmicb.2023.1164904 PMC10290204 37362945

[B13] Chen D. Zheng M. Zhou Y. Gao L. Zhou W. Wang M.. (2022). Improving the quality of Napier grass silage with pyroligneous acid: Fermentation, aerobic stability, and microbial communities. Front. Microbiol. 13. doi: 10.3389/fmicb.2022.1034198 PMC9745580 36523820

[B14] Cui Y. Liu H. Gao Z. Xu J. Liu B. Guo M.. (2022). Whole-plant corn silage improves rumen fermentation and growth performance of beef cattle by altering rumen microbiota. Appl. Microbiol. Biotechnol. 106, 4187–4198. doi: 10.1007/s00253-022-11956-5 35604439

[B15] Danner H. Holzer M. Mayrhuber E. Braun. R. (2003). Acetic acid increases stability of silage under aerobic conditions. Appl. Environ. Microbiol. 69 (1), 562–567. doi: 10.1128/aem.69.1.562-567.2003 12514042 PMC152389

[B16] Drouin P. Tremblay J. Renaud J. Apper. E. (2021). Microbiota succession during aerobic stability of maize silage inoculated with *Lentilactobacillus buchneri* NCIMB 40788 and *Lentilactobacillus hilgardii* CNCM-I-4785. Microbiologyopen 10 (1), e1153. doi: 10.1002/mbo3.1153 33369186 PMC7885010

[B17] Fazle Rabbee M. Baek K. H. (2020). Antimicrobial activities of lipopeptides and polyketides of *Bacillus velezensis* for agricultural applications. Molecules 25 (21), 4973. doi: 10.3390/molecules25214973 33121115 PMC7662345

[B18] Ferraretto L. F. Shaver R. D. Luck B. D. (2018). Silage review: Recent advances and future technologies for whole-plant and fractionated corn silage harvesting. J. Dairy Sci. 101, 3937–3951. doi: 10.3168/jds.2017-13728 29685271

[B19] Haq I. U. Sarwar M. K. Mohyuddin Z. (2021). Microbial determinants in silage rotting: a challenge in winter fodders. CRC Press 2021, 301–329. doi: 10.1201/9781003055365-15

[B20] Keshri J. Chen Y. Pinto R. Kroupitski Y. Weinberg Z. G. Sela Saldinger S. (2018). Microbiome dynamics during ensiling of corn with and without *Lactobacillus plantarum* inoculant. Appl. Microbiol. Biotechnol. 102, 4025–4037. doi: 10.1007/s00253-018-8903-y 29536147

[B21] Khalid F. Khalid A. Fu Y. Hu Q. Zheng Y. Khan S.. (2021). Potential of *Bacillus velezensis* as a probiotic in animal feed: a review. J. Microbiol. 59 (7), 627–633. doi: 10.1007/s12275-021-1161-1 34212287

[B22] Khan N. A. Yu P. Ali M. Cone J. W. Hendriks. W. H. (2015). Nutritive value of maize silage in relation to dairy cow performance and milk quality. J. Sci. Food Agric. 95 (2), 238–252. doi: 10.1002/jsfa.6703 24752455

[B23] Li D.-x. Ni K.-k. Zhang Y.-c. Lin Y.-l. Yang F.-y. (2018). Influence of lactic acid bacteria, cellulase, cellulase-producing Bacillus pumilus and their combinations on alfalfa silage quality. J. Integr. Agric. 17 (12), 2768–2782. doi: 10.1016/S2095-3119(18)62060-X

[B24] Li J. Wang W. Chen S. Shao T. Tao X. Yuan X. (2021). Effect of lactic acid bacteria on the fermentation quality and mycotoxins concentrations of corn silage infested with mycotoxigenic fungi. Toxins (Basel). 13 (10), 699. doi: 10.3390/toxins13100699 34678992 PMC8537395

[B25] Liu Y. Wang Z. Bao J. Si Q. Liu M. Jian Y.. (2023). Effect of carbohydrate on quality and aerobic stability of Leymus chinensis silage. Feed Res. (in chinese). 46 (16), 91–96. doi: 10.13557/j.cnki.issn1002-2813.2023.16.018

[B26] Lynch J. P. O’Kiely P. Waters S. M. Doyle. E. M. (2012). Conservation characteristics of corn ears and stover ensiled with the addition of *Lactobacillus plantarum* MTD-1, Lactobacillus plantarum 30114, or *Lactobacillus buchneri* 11A44. J. Dairy Sci. 95 (4), 2070–2080. doi: 10.3168/jds.2011-5013 22459852

[B27] McDonald P. Edwards R. A. Greenhalgh J. F. D. Morgan C. A. Sinclair L. A. Wilkinson R. G. (2010). Animal nutrition. 7th edition (Prentice Hall, UK).

[B28] Mu L. Xie Z. Hu L. Chen G. Zhang. Z. (2020). Cellulase interacts with *Lactobacillus plantarum* to affect chemical composition, bacterial communities, and aerobic stability in mixed silage of high-moisture amaranth and rice straw. Bioresour. Technol. 315, 123772. doi: 10.1016/j.biortech.2020.123772 32653750

[B29] Mu L. Xie Z. Hu L. Chen G. Zhang Z. (2021). *Lactobacillus plantarum* and molasses alter dynamic chemical composition, microbial community, and aerobic stability of mixed (amaranth and rice straw) silage. J. Sci. Food Agric. 101, 5225–5235. doi: 10.1002/jsfa.11171 33611793

[B30] Muck R. E. Nadeau E. M. G. McAllister T. A. Contreras-Govea F. E. Santos M. C. Kung L. Jr. (2018). Silage review: Recent advances and future uses of silage additives. J. Dairy Sci. 101 (5), 3980–4000. doi: 10.3168/jds.2017-13839 29685273

[B31] Nascimento Agarussi M. C. Gomes Pereira O. Paula R. A. Silva V. P. D. Santos Roseira J. P. Fonseca E.. (2019). Novel lactic acid bacteria strains as inoculants on alfalfa silage fermentation. Sci. Rep. 9 (1), 8007. doi: 10.1038/s41598-019-44520-9 31142784 PMC6541639

[B32] Ning T. Wang H. Zheng M. Niu D. Zuo S. Xu. C. (2017). Effects of microbial enzymes on starch and hemicellulose degradation in total mixed ration silages. Asian-Australas J. Anim. Sci. 30 (2), 171–180. doi: 10.5713/ajas.16.0046 27165015 PMC5205603

[B33] Pahlow G. Muck R. E. Driehuis F. Elferink S. J. W. H. O. Spoelstra S. F. (2003). “Microbiology of ensiling,” Silage Science and Technology, eds Buxton D.R. Muck R.E. Harrison J.H.. doi: 10.2134/agronmonogr42.c2

[B34] Reed H. Mueller B. Groves C. L. Smith. D. L. (2022). Presence and correlation of *Fusarium graminearum* and *Deoxynivalenol accumulation* in silage corn plant parts. Plant Dis. 106 (1), 87–92. doi: 10.1094/pdis-03-21-0641-re 34491093

[B35] Richard J. L. (2007). Some major mycotoxins and their mycotoxicoses—An overview. Int. J. Food Microbiol. 119, 3–10. doi: 10.1016/j.ijfoodmicro.2007.07.019 17719115

[B36] Shi R. Li S. Wang Y. Jiang G. Zhang Y. Li W. (2022a). Current situation and thinking of promoting whole plant silage corn. Modernizing Agric. (in chinese). 2022 (11), 26–28.

[B37] Shi Z. Xiao Q. Yu Z. (2022b). Advances in aerobic stability of whole plant corn silage. Feed Industry Magazine 43, 14. doi: 10.13302/j.cnki.fi.2022.23.003

[B38] Silva T. B. P. Del Valle T. A. Ghizzi L. G. Silva G. G. Gheller L. S. Marques J. A.. (2021). Partial replacement of corn silage with whole-plant soybean and black oat silages for dairy cows. J. Dairy Sci. 104, 9842–9852. doi: 10.3168/jds.2021-20200 34099291

[B39] Tabbene O. Ben Slimene I. Bouabdallah F. Mangoni M. L. Urdaci M. C. Limam. F. (2009). Production of anti-methicillin-resistant Staphylococcus activity from Bacillus subtilis sp. strain B38 newly isolated from soil. Appl. Biochem. Biotechnol. 157 (3), 407–419. doi: 10.1007/s12010-008-8277-1 18712291

[B40] Van Soest P. J. Robertson J. B. Lewis. B. A. (1991). Methods for dietary fiber, neutral detergent fiber, and nonstarch polysaccharides in relation to animal nutrition. J. Dairy Sci. 74 (10), 3583–3597. doi: 10.3168/jds.S0022-0302(91)78551-2 1660498

[B41] Wang C. He L. Xing Y. Zhou W. Yang F. Chen X.. (2019). Fermentation quality and microbial community of alfalfa and stylo silage mixed with Moringa oleifera leaves. Bioresour Technol. 284, 240–247. doi: 10.1016/j.biortech.2019.03.129 30947138

[B42] Wang Y. Ke W. Lu Q. Zhang. G. (2023). Effects of *Bacillus coagulans* and *Lactobacillus plantarum* on the Fermentation Characteristics, Microbial Community, and Functional Shifts during Alfalfa Silage Fermentation. Anim. (Basel). 13 (5). doi: 10.3390/ani13050932 PMC10000087 36899789

[B43] Wilkinson J. M. Bolsen K. K. Lin C. J. (2003). “History of silage,” in Silage Science and Technology, eds Buxton D.R. Muck R.E. Harrison J.H.. doi: 10.2134/agronmonogr42.c1

[B44] Wu B. Hu Z. Wei M. Yong M. Niu H. (2022). Effects of inoculation of *Lactiplantibacillus plantarum* and *Lentilactobacillus buchneri* on fermentation quality, aerobic stability, and microbial community dynamics of wilted Leymus chinensis silage. Front. Microbiol. 13. doi: 10.3389/fmicb.2022.928731 PMC9372395 35966710

[B45] Xu D. Ding W. Ke W. Li F. Zhang P. Guo. X. (2018). Modulation of metabolome and bacterial community in whole crop corn silage by inoculating homofermentative *Lactobacillus plantarum* and heterofermentative *Lactobacillus buchneri* . Front. Microbiol. 9. doi: 10.3389/fmicb.2018.03299 PMC6352740 30728817

[B46] Xu D. Wang N. Rinne M. Ke W. Weinberg Z. G. Da M.. (2021). The bacterial community and metabolome dynamics and their interactions modulate fermentation process of whole crop corn silage prepared with or without inoculants. Microb. Biotechnol. 14 (2), 561–576. doi: 10.1111/1751-7915.13623 32627363 PMC7936295

[B47] Yang F. Wang Y. Zhao S. Feng C. Fan. X. (2021). Dynamics of the fermentation products, residual non-structural carbohydrates, and bacterial communities of wilted and non-wilted alfalfa silage with and without *Lactobacillus plantarum* inoculation. Front. Microbiol. 12. doi: 10.3389/fmicb.2021.824229 PMC8788936 35087507

[B48] Zhang Y. Tao X. Liu Q. Zhang Y. J. Xu J. Zhang W.. (2022b). Succession changes of fermentation parameters, nutrient components and bacterial community of sorghum stalk silage. Front. Microbiol. 13. doi: 10.3389/fmicb.2022.982489 PMC9386229 35992672

[B49] Zhang H. Zhang L. Xue X. Zhang X. Wang H. Gao T.. (2022a). Effect of feeding a diet comprised of various corn silages inclusion with peanut vine or wheat straw on performance, digestion, serum parameters and meat nutrients in finishing beef cattle. Anim. Biosci. 35, 29–38. doi: 10.5713/ab.21.0088 34237922 PMC8738933

[B50] Zhao S. Yang F. Wang Y. Fan X. Feng C. Wang. Y. (2021). Dynamics of fermentation parameters and bacterial community in high-moisture alfalfa silage with or without lactic acid bacteria. Microorganisms 9 (6), 1225. doi: 10.3390/microorganisms9061225 34200084 PMC8226466

[B51] Zhao X. Y. Zhang X. F. Song L. W. Zhu C. X. Bao J. P. Gao M.. (2020). Effects of whole corn silage on growth performance, slaughter performance and meat quality of mutton sheep. Chin. J. Anim. Nutr. 32, 253–258. doi: 10.3969/j.issn.1006-267x.2020.01.031

[B52] Zhu Y. Xiong H. Wen Z. Tian H. Chen Y. Wu L.. (2022). Effects of different concentrations of *lactobacillus plantarum* and *bacillus licheniformis* on silage quality, in *vitro* fermentation and microbial community of hybrid pennisetum. Anim. (Basel). 12 (14), 1752. doi: 10.3390/microorganisms9061225 PMC9311531 35883299

